# Morphology and Molecular Identification of Twelve Commercial Varieties of Kiwifruit

**DOI:** 10.3390/molecules24050888

**Published:** 2019-03-03

**Authors:** Qiaoli Xie, Hongbo Zhang, Fei Yan, Chunxia Yan, Shuguang Wei, Jianghua Lai, Yunpeng Wang, Bao Zhang

**Affiliations:** 1 School of Forensic Medicine, Xi’an Jiaotong University, 76 Yanta West Road, Xi’an 710061, China; sunshineqiaoer@xjtu.edu.cn (Q.X.); zhanghb@mail.xjtu.edu.cn (H.Z.); yanchunxia@mail.xjtu.edu.cn (C.Y.); weisg@mail.xjtu.edu.cn (S.W.); laijh1011@mail.xjtu.edu.cn (J.L.); 2 School of Energy and Power Engineering, Chongqing University, 174 Shapingba Main Street, Chongqing 400030, China; yanfei0506@cqu.edu.cn

**Keywords:** food safety, kiwifruit (*Actinidia chinensis*), molecular identification, phylogeny, DNA barcode

## Abstract

The quality and safety of food are important guarantees for the health and legal rights of consumers. As an important special fruitcrop, there are frequently shoddy practices in the kiwifruit (*Actinidia chinensis*) market, which harms the interests of consumers. However, there is lack of rapid and accurate identification methods for commercial kiwifruit varieties. Here, twelve common commercial varieties of kiwifruit were morphologically discriminated. DNA barcodes of chloroplast regions *psbA-trnH*, *rbcL*, *matK*, *rpoB*, *rpoC1*, *ycf1b*, *trnL* and *rpl32_trnL*(*UAG*), the nuclear region *At103* and intergenic region *ITS2* were amplified. Divergences and phylogenetic trees were used to analyze the phylogenetic relationship of these twelve commercial kiwifruit varieties. The results showed that *matK*, *ITS2* and *rpl32_trnL*(*UAG*) can be utilized as molecular markers to identify CuiYu, JinYan, HuangJinGuo, ChuanHuangJin, HuaYou, YaTe, XuXiang and HongYang. This provides experimental and practical basis to scientifically resolve kiwifruit-related judicial disputes and legal trials.

## 1. Introduction

The quality and safety of food are important guarantees for the health and legal rights of consumers. Kiwifruit (*Actinidia chinensis*), also called ‘the king of fruits’, is an important economical crop because of its exceedingly high content of ascorbic acid (vitamin C), dietary fiber, nutritional minerals compositions and other health beneficial metabolites [1]. China, the origin of kiwifruit, possesses the largest planted area of kiwifruit in the world. According to statistics, in 2016, the national kiwifruit cultivation area reached 0.365 million acres, and the output was 2.15 million tons (China Industry Report Network) [2] Since the commercial establishment of kiwifruit, its classification has been controversial. These kiwifruits, named with geographical indications, cannot represent the species of kiwifruit. Besides, many commercial varieties vary greatly in market demand and price due to differences in taste and nutritional value. Therefore, there are frequent problems in the kiwifruit industry, such as false labelling, and lack of origin confirmation and identification. In order to protect consumer rights, we are looking for ways to quickly and accurately identify the commercial varieties of kiwifruit in the market and scientifically resolve judicial disputes and legal trials. However, morphological-based identification methods have great difficulties for non-professionals, and the methods of omics or chemistry are complex, time consuming and susceptible to environmental factors [3,4].

The DNA barcoding technique is a quick and effective molecular marker technology for classification and identification of organisms by using a standard gene region [5]. Since Hebert et al. recommended mitochondrial *cox1* gene as a DNA barcode for animal species identification in 2003 [5], it has been widely and effectively applied in the classification, identification, and phylogenetic analysis of thousands of species [6,7]. DNA barcode technology is an ideal identification method because of its accurate identification and simple operation. To date, the Consortium for the Barcode of Life (CBOL) formally proposed chloroplast markers *rbcL* and *matK* as the core barcodes for plant species identification [8]. Noncoding intergenic spacer *psbA-trnH* barcode was used to identify species of medicinal pteridophytes and members of Dendrobium of Orchid [9,10]. *ITS2* was selected as a standard barcode for identifying medical plants [11]. Two chloroplast genome markers, coding *rpoB* and *rpoC1* were utilized to discriminate 92 species in 32 diverse genera of land plants [12]. *ycf1b* was reported as the most variable plastid genome region and can serve as a core barcode of land plants [13]. *trnL* was chosen as the barcoding gene for reference library constructing and high-throughput sequencing for wetland plants [14]. *At103* (Mgprotoporphyrin IX monomethyl ester cyclase) developed by Li et al. [15] as universally amplifiable marker for phylogenetic reconstructions and that together with *matK* to be used to distinguish toxic hybrids form parental species [16]. Fu et al. [17] found that *rpl32_trnL* (*UAG*) had a greater degree of variation and could be used as the core barcode sequence of cherry plants. These genes have the potential to be as powerful as mitochondrial CO1 gene in identifying species [10]. However, as far as we have been concerned, there have been no reports on DNA barcode for common kiwifruit commercial varieties in the market. Previous studies have provided us with reference to the feasibility of using DNA barcodes to identify kiwifruit commercial varieties. Lee et al. made use of *ITS2* to identify the varieties and provenances of Taiwan’s domestic and imported made teas [18]. Enan and Ahmed using chloroplast DNA barcode *psbK-psbI* spacers for identification of Emirati date palm (*Phoenix dactylifera* L.) varieties (cultivar-level) [19]. Jaakola et al. successfully identified the blueberry varieties “Northcountry” and “Northblue” using DNA barcode technology combined with high-resolution dissolution profiles [20]. Through DNA barcodes, He et al. authenticated cultivars of *Angelica anomala* Ave’-Lall [21]. 

Single nucleotide polymorphism (SNP) is a single nucleotide variation with a specific and determined genetic location in at least 1% of the population [22]. SNP is one of the richest and most stable genetic polymorphisms in the genome, which is suitable for solving the differences between closely related species [23]. SNP typing has been successfully used to conveniently and accurately identify plant origin [24], medicinal plants [25], and bacteria [26].

In this study, we attempted to use the morphological method combined with molecular biological methods for rapid cultivar identification of twelve kiwifruit commercial varieties in China. SNP typing method was evaluated by using ten candidate DNA barcoding markers (chloroplast genome *psbA-trnH*, *rbcL*, *matK*, *rpoB*, *rpoC1*, *ycf1b*, *trnL* and *rpl32_trnL*(*UAG*), the nuclear region *At103* and intergenic region *ITS2*), which were used for molecular identification. This study will not only lay a foundation for phylogenetic analysis but also provides experimental and practical basis for the rapid identification of kiwifruit.

## 2. Results

### 2.1. Morphological Identification

**Figure 1:**
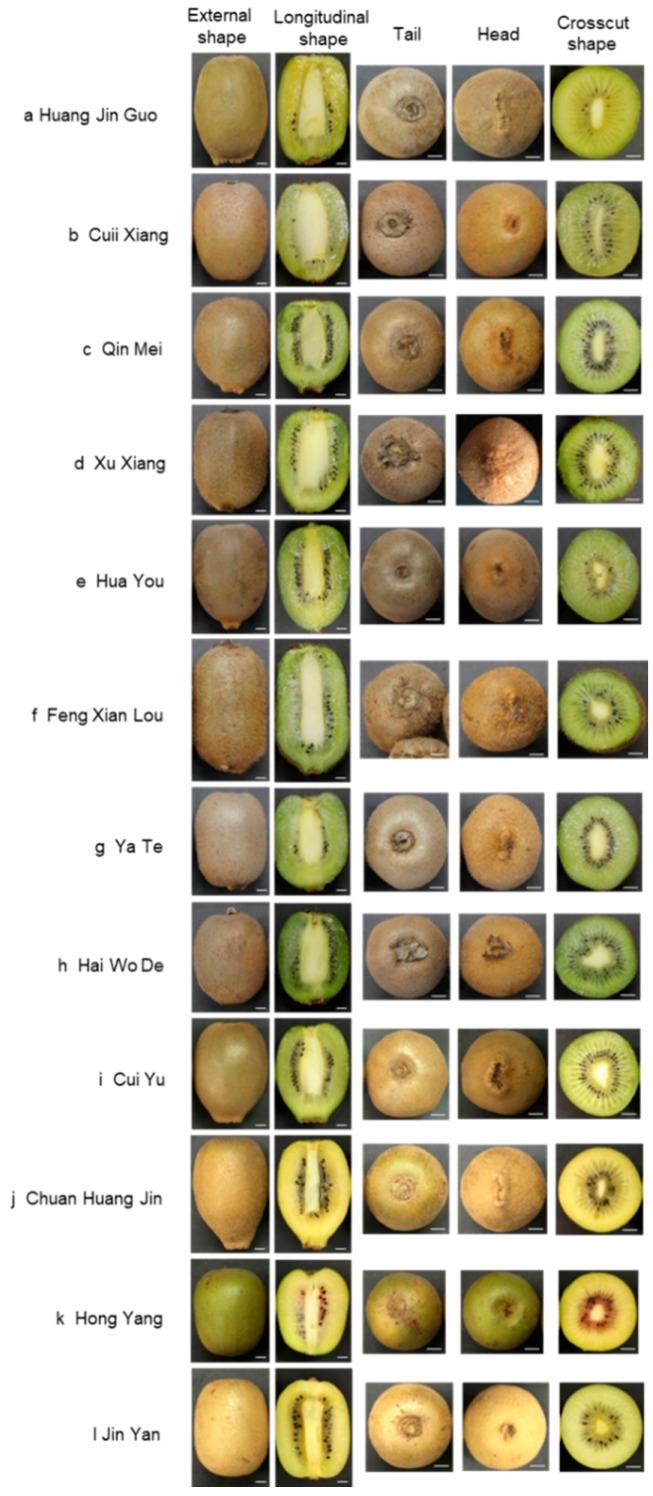
External and internal forms of twelve commercial kiwifruit. (**a**) HuangJinGuo, (**b**) CuiXiang, (**c**) QinMei, (**d**) XuXiang, (**e**) HuaYou, (**f**) FengXianLou, (**g**) YaTe, (**h**) HaiWoDe, (**i**) CuiYu, (**j**) ChuanHuangJin, (**k**) HongYang, (**l**) JinYan. Each variety contains external shape, longitudinal shape, head, tail, crosscut shape. Scale bar represents 6 mm.

In order to enable consumers to visually identify the commercial varieties of kiwifruit in the first place, by referring to the classification criteria of “Flora of China”, the morphology of 12 commercial kiwifruit were analyzed. We mainly made statistics on fruit type, fruit shape, fruit size, peel color, peel spots, hair presence, hair length, hair softness and hardness, hair shedding, pulp color, pulp taste, seed color, seed number and shape, beak prominence, beak diameter, fruit picking time and so on. The results are shown in Figure 1 and Supplementary Table S1. The 72 samples of the 12 commercial varieties of kiwifruit were harvested from 150–160 days after pollination. Their fruits are all bacca. The shape of the fruit is mostly cylindrical and spherical. HuangJinGuo and ChuanHuangJin are long oval. JinYan is cylindrical. HongYang is short cylindrical and CuiYu is oblate cone. Statistical analysis of fruit size showed that the fruit size of FengXianLou is the largest, followed by HuangJinGuo, CuiXiang, and HongYang is the smallest. For the peel, only CuiYu has no spots on the peel surface, other commercial varieties all have spots. The color of mostly commercial varieties’ peel is brown. The fruit peel of HuangJinGuo, ChuanHuangJin and JinYan is yellower. The peel of HongYang is greenish. Hair analysis found that the skin of HuangJinGuo, ChuanHuangJin, CuiYu and HongYang have no hair, other commercial varieties all have hair. QinMei and HaiWoDe have very dense hair on the surface. HuaYou has few hair on the surface. The surface of FengXianLou has the longest hair, soft and easy to fall off. HuaYou’s surface hair is the shortest and easy to fall off. 

All statistical results are in Supplementary Table S1. For the color of the flesh, the HongYang is the easiest to distinguish, which has a radial red color in the pulp center. The flesh color of HuangJinGuo, ChuanHuangJin and JinYan are yellow. The flesh color of HuaYou is yellow-green and other commercial varieties are all green. The fruit of HuangJinGuo, ChuanHuangJin, JinYan, CuiXiang, HuaYou, CuiYu are sweet, no sour taste, and other commercial varieties are sweet and sour. Most of the seeds are dark brown and flat oval. HaiWoDe has the largest number of seeds, and HuangJinGuo has the fewest seeds. Statistical results are in Supplementary Table S1. Kiwifruit has a beak at the top. Except CuiXiang and HongYang, the beaks of other commercial varieties are prominent. The longest beak exists on HuangJinGuo, followed by QinMei, and HongYang has the smallest beak.

The above analysis shows that HongYang is the most discernible variety by the peel color and flesh color. JinYan is easy to identify because of its yellow peel and flesh. The shape of CuiYu is similar to that of HuangJinGuo, while there are differences between them. The surface of CuiYu has no spots and its flesh is green. The surface of HuangJinGuo is spotted and its flesh is yellow. Therefore, CuiYu is easier to be distinguished. The hair of Huayou is short and small and its flesh is yellow-green, which made it easier to be discriminated. FengXianLou is a long cylindrical shape with many hairs easy to fall off makes it easy to be identified. It is not easy to distinguish HuangJinGuo and ChuanHuangJin. CuiXiang, QinMei, XuXiang, YaTe and HaiWoDe all have green pulp, but the shape of CuiXiang is slightly flat, and its beak is the smallest and not prominent. CuiXiang is sweet, while QinMei, XuXiang, YaTe and HaiWoDe are sour and sweet. YaTe’s skin is white and brown compared with QinMei, XuXiang and HaiWoDe. QinMei is greener than XuXiang and HaiWoDe. For ordinary consumers, XuXiang and HaiWoDe are relatively difficult to be differentiated.

### 2.2. Analysis of Variable Sites in Different Commercial Varieties

**Figure 2:**
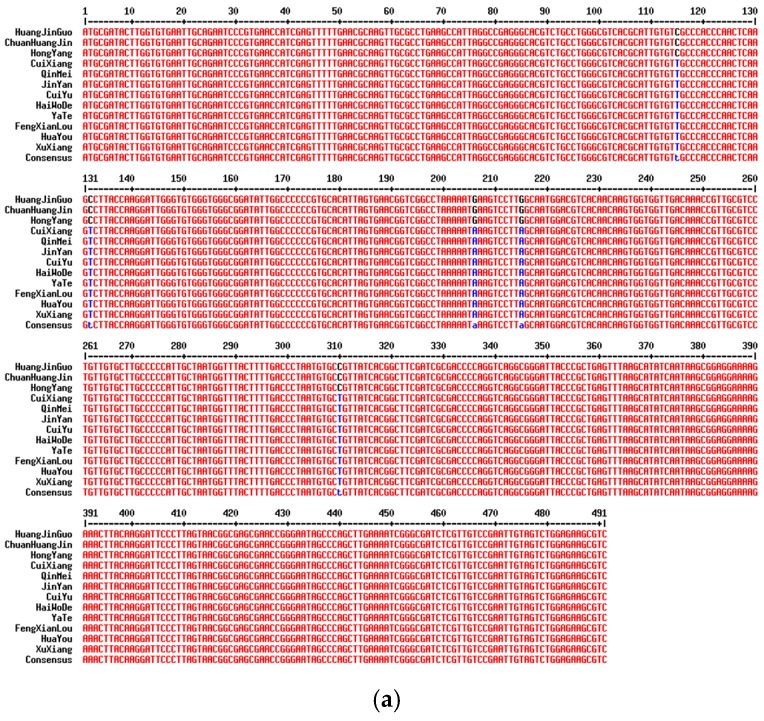

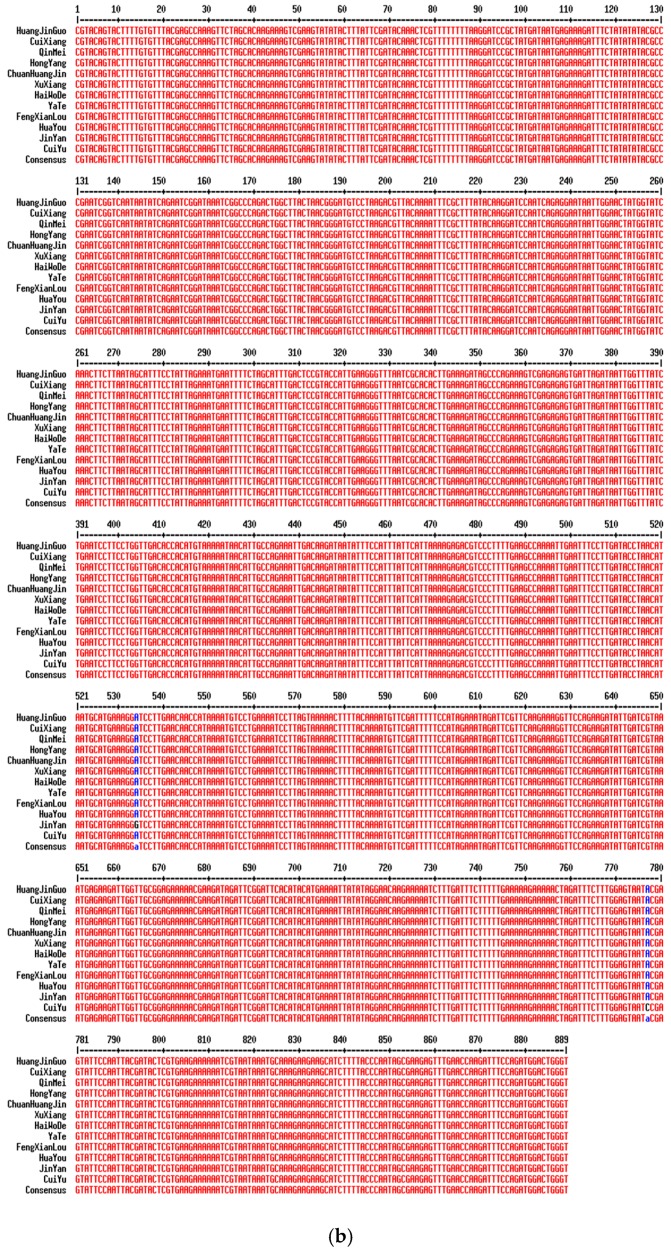

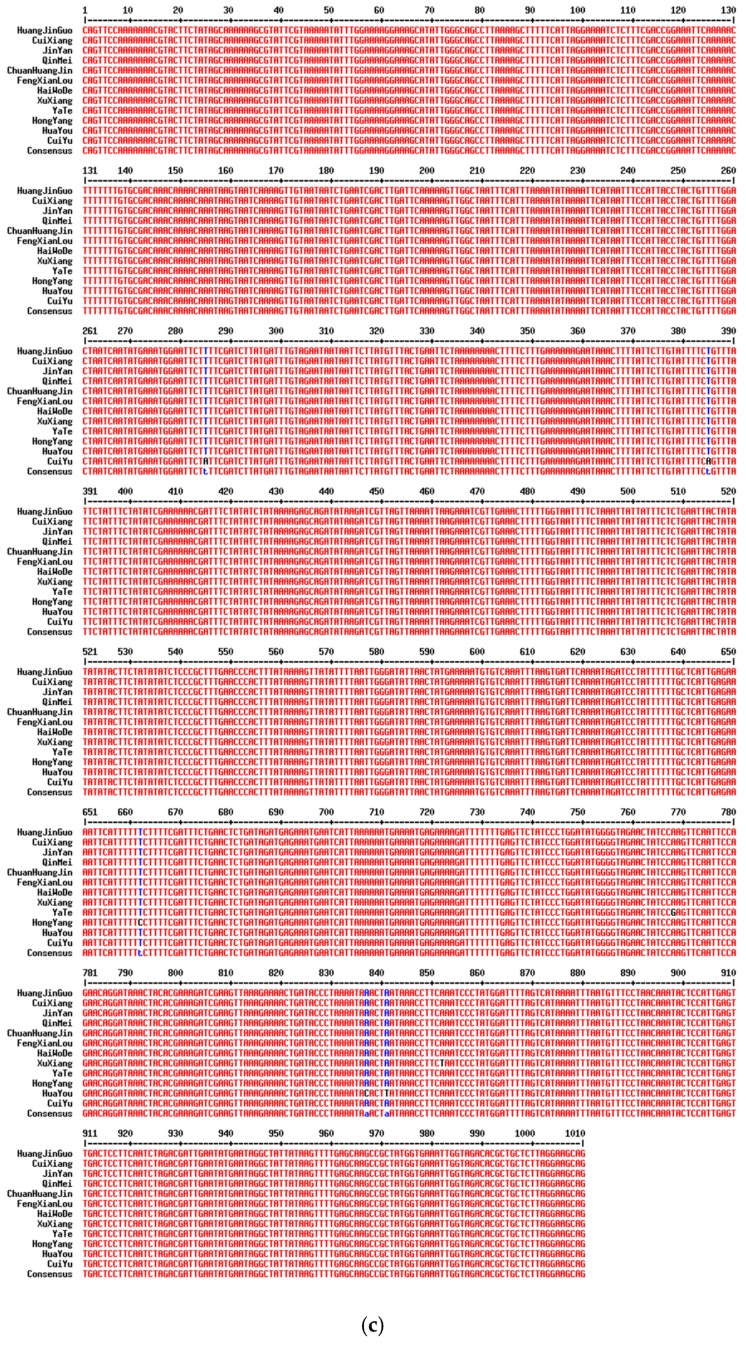
DNA barcode sequence alignment of 12 commercial kiwifruits. (**a**) *ITS2* sequence alignment 491 bp; (**b**) *matK* sequence alignment 889 bp; (**c**) *rpl32_trnL*(*UAG*) sequence alignment 1010 bp.

The sequencing results showed that the product length of *rbcL*, *matK*, *psbA-trnH*, *ITS2*, *rpoB, rpoC1*, *trnL* and *rpl32_trnL*(*UAG*) are 743 bp, 889 bp, 502 bp, 491 bp, 512 bp, 529 bp, 193bp and 1010 bp, respectively. Sequences of *rbcL*, *psbA-trnH*, *rpoB*, *rpoC1* and *trnL* have no difference in these twelve kiwi commercial varieties (Supplementary Figure S1). *ycf1b* and *At103* get no results of amplification. The amplification efficiency of 72 *ITS2* sequences is 100%. Sequencing results showed that the sequences of 6 repetitive samples from each commercial variety are consistent and stable. There are two haplotypes in *ITS2* sequence. Bases at 115 bp, 132 bp and 310 bp of HuangJinGuo, ChuanHuangJin and HongYang are “C”, that of the other nine commercial varieties are “T”. Bases at 206 bp and 215 bp of HuangJinGuo, ChuanHuangJin and HongYang are “G”, that of the other nine commercial varieties are “A” (Figure 2a, Table 1 and Supplementary Table S2). 

For *matK*, the amplification efficiency of 72 *matK* sequences is 100%. Sequencing results showed that the sequences of 6 repetitive samples of each commercial variety are consistent and stable. There are three haplotypes in *matK* sequences. Bases at 534 bp of JinYan are “G”, which of the other eleven commercial varieties are “A”, and bases at 777 bp of CuiYu are “C”, the other eleven commercial varieties are “A” (Figure 2b, Table 1 and Supplementary Table S3). The amplification efficiency of 72 *rpl32_trnL*(*UAG*) sequences is 100%. There are six haplotypes in *rpl32_trnL*(*UAG*) sequence. Bases at 285 bp and 385 bp of CuiYu are “A”, that of the other 11 commercial varieties are “T”. Bases at 662 bp of HongYang are “C”, that of the other 11 commercial varieties are “T”. Bases at 768 bp of YaTe are “G”, that of the other 11 commercial varieties are “A”. Bases at 837 bp and 841 bp of HuaYou are “C” and “T”, respectively, that of the other 10 commercial varieties are “A”. Bases at 852 bp of XuXiang are “T”, that of the other 11 commercial varieties are “A”. (Figure 2c, Table 1 and Supplementary Table S4). 

### 2.3. K2P Genetic Distance Analysis

Genetic distances of all samples were calculated by using the MEGA 7.0 [27] software (Pennsylvania State University, USA). The results of *ITS2*, *matK*, *rpl32_trnL*(*UAG*) and *ITS2*+*matK*+*rpl32_trnL*(*UAG*) are shown in Supplementary Table S6. For *ITS2*, there is no difference among HuangJinGuo, ChuanHuangJin and HongYang. The genetic distance between them and nine other commercial varieties is 0.01. There is no difference among the nine commercial varieties (Supplementary Table S5a). For *matK*, the genetic distance between JinYan and CuiYu is 0.002, and between JinYan and other 10 commercial varieties is 0.001. The genetic distance between CuiYu and other 10 cultivars is 0.001, and there is no difference among the other 10 cultivars (Supplementary Table S5b). For *rpl32_trnL*(*UAG*), the genetic distance between HuaYou and HongYang, YaTe, XuXiang is 0.003. The genetic distance between HuaYou and JinYan, CuiXiang, FengXianLou, ChuanHuangJin, HaiWoDe, QinMei and HuangJinGuo is 0.002. The genetic distance between HongYang and CuiYu is 0.003. The genetic distance between HongYang and YaTe, XuXiang is 0.002. The genetic distance between HongYang and JinYan, CuiXiang, FengXianLou, ChuanHuangJin, HaiWoDe, QinMei and HuangJinGuo is 0.001. The genetic distance between CuiYu and YaTe, XuXiang is 0.003. The genetic distance between CuiYu and JinYan, CuiXiang, FengXianLou, ChuanHuangJin, HaiWoDe, QinMei and HuangJinGuo is 0.001. The genetic distance between JinYan and YaTe, XuXiang is 0.001. There is no difference between JinYan and CuiXiang, FengXianLou, ChuanHuangJin, HaiWoDe, QinMei and HuangJinGuo. The genetic distance between CuiXiang and YaTe, XuXiang is 0.001. There is no difference between CuiXiang and FengXianLou, ChuanHuangJin, HaiWoDe, QinMei and HuangJinGuo. The genetic distance between YaTe and XuXiang is 0.002. There is no difference between YaTe and FengXianLou, ChuanHuangJin, HaiWoDe, QinMei and HuangJinGuo. The genetic distance between FengXianLou and XuXiang is 0.001. There is no difference between FengXianLou and ChuanHuangJin, HaiWoDe, QinMei and HuangJinGuo. The genetic distance between ChuanHuangJin and XuXiang is 0.001. There is no difference between ChuanHuangJin and HaiWoDe, QinMei and HuangJinGuo. The genetic distance between HaiWoDe and XuXiang is 0.001. There is no difference between HaiWoDe and QinMei and HuangJinGuo. The genetic distance between QinMei and XuXiang is 0.001. There is no difference between QinMei and HuangJinGuo. The genetic distance between HuangJinGuo and XuXiang is 0.001 (Supplementary Table S5c). For *ITS2*+*matK*+*rpl32_trnL*(*UAG*), the genetic distance between YaTe and JinYan is 0.001. The genetic distance between XuXiang and YaTe, JinYan is 0.001. There is no difference between QinMei and YaTe, JinYan, XuXiang. There is no difference between HaiWoDe and YaTe, JinYan, XuXiang, QinMei. There is no difference between CuiXiang and YaTe, JinYan, XuXiang, QinMei, HaiWoDe. The genetic distance between HuaYou and YaTe, JinYan, XuXiang, QinMei, HaiWoDe, CuiXiang is 0.001. There is no difference between FengXianLou and YaTe, JinYan, XuXiang, QinMei, HaiWoDe and CuiXiang. The genetic distance between FengXianLou and HuaYou is 0.001. The genetic distance between CuiYu and YaTe, JinYan, XuXiang, HuaYou is 0.002. The genetic distance between CuiYu and QinMei, HaiWoDe, CuiXiang, FengXianLou is 0.001. The genetic distance between HuangJinGuo and YaTe, JinYan, XuXiang, HuaYou, CuiYu is 0.003. The genetic distance between HuangJinGuo and QinMei, HaiWoDe and CuiXiang, FengXianLou is 0.002. The genetic distance between HongYang and YaTe, JinYan, XuXiang, QinMei, HaiWoDe, CuiXiang, HuaYou, FengXianLou is 0.003. The genetic distance between HongYang and CuiYu is 0.004. There is no difference between HongYang and CuiYu. The genetic distance between ChuanHuangJin and YaTe, JinYan, XuXiang, HuaYou, CuiYu is 0.003. The genetic distance between ChuanHuangJin and QinMei, HaiWoDe and CuiXiang, FengXianLou is 0.002. There is no difference between ChuanHuangJin and HuangJinGuo, HongYang (Supplementary Table S5d).

### 2.4. Phylogenetic Analysis

**Figure 3:**
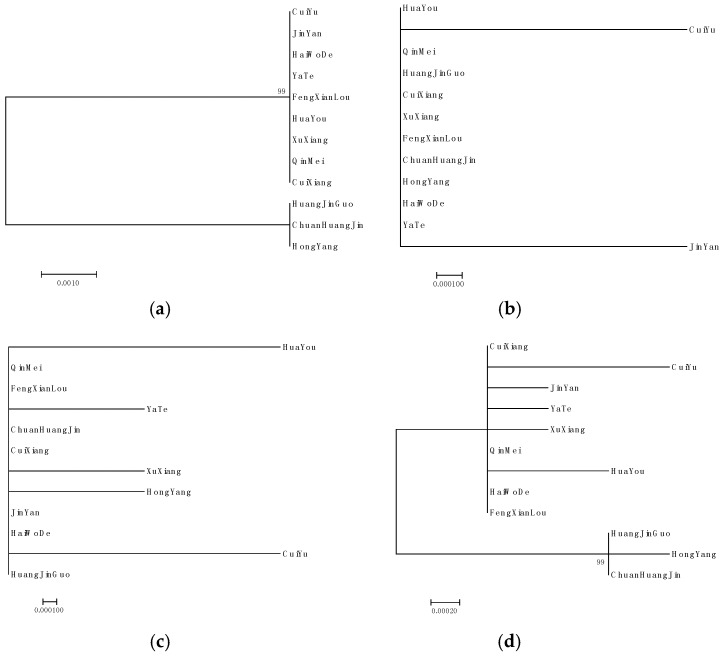
Phylogenetic analysis of 12 kiwifruit commercial varieties. (**a**) Analysis of *ITS2* sequence fragments; (**b**) Analysis of *matK* sequence fragments; (**c**) Analysis of *rpl32_trnL*(*UAG*) sequence fragments; (**d**) Analysis of *ITS2+matK+rpl32_trnL*(*UAG*) sequence fragments.

*ITS2*, *matK* and *rpl32_trnL*(*UAG*) sequences and *ITS2*+*matK*+*rpl32_trnL*(*UAG*) combination sequences were tested 1000 times by bootstrap method to build NJ phylogenetic tree (Figure 3 and Supplementary Figure S2). Phylogenetic analysis of *ITS2* gene sequences (Figure 3a and Supplementary Figure S2a) showed that HuangJinGuo, ChuanHuangJin and HongYang were clustered into one group, and the other nine commercial varieties were grouped into another category. For the *matK* gene sequences, JinYan and CuiYu were separately distinguished, and the other ten commercial varieties were clustered into one category (Figure 3b and Supplementary Figure S2b). Figure 3c and Supplementary Figure S2c indicated that, using *rpl32_trnL*(*UAG*), HuaYou, YaTe, XuXiang, HongYang, CuiYu could be distinguished from the other seven commercial varieties, respectively. 

For *ITS2*+*matK*+*rpl32_trnL*(*UAG*), CuiYu, JinYan, YaTe, XuXiang, HuaYou, HongYang, HuangJinGuo and ChuanHuangJin can be distinguished from the other four commercial varieties, respectively(Figure 3d).

## 3. Discussion

The accurate classification of kiwifruit is one of the problems in the current kiwifruit plant resource utilization and market regulation. It is very difficult to classify kiwifruit only by morphology, because many shapes of kiwifruit are very close that requires the participation of professionals. If the suitable DNA barcode can be screened for kiwifruit, it is very meaningful to classification and identification, resource protection, variety selection and market regulation of kiwifruit.

In this study, 72 samples of 12 commercial varieties were collected in the market. We firstly conducted a morphological analysis of twelve kiwifruit commercial varieties that are easily confused in the market. Suggestions for morphological identification are proposed. By referring to the classification criteria of “Flora of China (http://foc.iplant.cn/)”, the morphology of 12 commercial kiwifruit was analyzed in Supplementary Table S1. We mainly made statistics on fruit type, fruit shape, fruit size, peel color, peel spots, hair presence, hair length, hair softness and hardness, hair shedding, pulp color, pulp taste, seed color, seed number and shape, beak prominence, beak diameter, fruit picking time (Supplementary Table S1). The analysis results showed that HongYang is most discernible by the peel color and flesh color. Therefore, the expensive HongYang, whose price is 4 to 5 times than that of QinMei, HuaYou, YaTe and HaiWoDe in the market, can be identified morphologically. JinYan whose price is 3 to 4 times than that of QinMei, HuaYou, YaTe and HaiWoDe in the market, is easy to identify because of its yellow peel and flesh. The shape of CuiYu is similar to that of HuangJinGuo, while there are differences between them. The surface of HuangJinGuo is spotted and its flesh is yellow. Therefore, CuiYu is easier to be distinguished. The hair of Huayou is short and small and its flesh is yellow-green, which made it easier to be discriminated. FengXianLou is a long cylindrical shape with many hairs easy to fall off makes it easy to be identified. It is not easy to distinguish HuangJinGuo and ChuanHuangJin. CuiXiang, QinMei, XuXiang, YaTe and HaiWoDe all have green pulp, but the shape of CuiXiang is slightly flat, and its beak is the smallest and not prominent. CuiXiang is sweet, while QinMei, XuXiang, YaTe and HaiWoDe are sour and sweet. YaTe’s skin is white and brown compared with QinMei, XuXiang and HaiWoDe. QinMei is greener than XuXiang and HaiWoDe. For ordinary consumers, XuXiang and HaiWoDe are relatively difficult to be differentiated. As the identification of morphology is very demanding on the professional, there are limitations in the identification of morphology. 

Recently, DNA barcodes are often used as a standard for identifying plant species [28,29]. The DNA region, as an effective barcode, should contain sufficient variability for identification, contain conserved regions for the development of universal primers and be short enough to be sequenced in one reaction. In animals, a portion of mitochondrial cytochrome C oxidation enzyme I gene sequence is often used as a general barcode and is also used in forensic identification [16,28]. Currently, there is no common area available in plants. In the nuclear and plastid genomes, there are multiple loci were selected as DNA barcodes in plants [12,30,31,32].

Ten DNA barcoding *rbcL*, *matK*, *psbA-trnH*, *rpoB*, *rpoC1, ITS2*, *ycf1b*, *trnL*, *rpl32_trnL*(*UAG*) and *At103* were explored to quickly and accurately identify twelve kiwifruit commercial varieties in this study. The primer sequences used for amplification of these ten regions, the length of the products, and the amplification procedures are listed in Table 2. The *psbA-trnH* sequence of the intergenic region is one of the most mutated non-coding regions in the plant chloroplast genome and is often used as a barcode for material identification [32,33,34]. However, the results of this experiment found that the *psbA-trnH* sequences of the 12 commercial kiwifruits are identical, which means that the *psbA-trnH* is not suitable for identifying the 12 commercial kiwifruits. Intergenic region *ITS2* (internal transcribed spacer 2) and the cp gene *rbcL* may have potential as universal plant barcodes [34]. Three regions *matK*, *rpoB* and *rpoC1* were outlined as viable markers for land plant barcoding [33]. CBOL formally proposed chloroplast markers *rbcL* and *matK* as the core barcodes for plant species identification [8]. The results, in this study, showed that the *rbcL*, *rpoB* and *rpoC1* sequences of the 12 commercial kiwifruits are unanimous, which indicates the three barcodes sequences are improper for the identification of the 12 commercial kiwifruits. *ycf1b* was reported as the most variable plastid genome region and can serve as a core barcode of land plants [13]. *At103* together with *matK* to be used to distinguish toxic hybrids form parental species [16]. Our results showed that these two DNA barcodes could not be amplified in the 12 commercial varieties of kiwifruit, indicating they are not suitable to be DNA barcodes of kiwifruit. *trnL* was chosed as the barcoding gene for reference library constructing for wetland plants [14], whose amplification results were indistinguishable among the 12 commercial varieties of kiwifruit. Therefore, *trnL* could not be used as the barcode to discriminate these 12 commercial varieties of kiwifruit.

There are two SNP sites in the *matK* sequences of 12 commercial kiwifruits. For *matK*, bases at 534 bp of Jin Yan are “G”, that of the other eleven commercial varieties are “A”, and bases at 777 bp of Cui Yu are “C”, that of the other eleven commercial varieties are “A” (Figure 2b, Table 1 and Supplementary Table S3). It can be applied to distinguish Jin Yan, CuiYu and other 10 commercial varieties. Genetic distance analysis discovered that the genetic distance between JinYan and CuiYu is 0.002, and between JinYan and 10 other commercial varieties is 0.001. The genetic distance between CuiYu and other 10 cultivars is 0.001, and there is no difference among the other 10 cultivars (Supplementary Table S5b). The phylogenetic analysis also found that Jin Yan, CuiYu and other ten commercial varieties is clustered into three branches. JinYan and CuiYu is separately distinguished from other ten commercial varieties, respectively (Figure 3b).

In addition, *ITS2* sequence analysis of 12 commercial kiwifruit results showed that compared to *rbcL*, *matK*, *psbA-trnH*, *rpoB*, *rpoC1* and *trnL*, the *ITS2* sequence difference is better. It was found that there are five SNPs in the 491 bp of *ITS2* sequence, bases at 115 bp, 132 bp and 310 bp of HuangJinGuo, ChuanHuangJin and HongYang are “C”, that of the other nine commercial varieties are “T”. Bases at 206 bp and 215 bp of HuangJinGuo, ChuanHuangJin and HongYang are “G”, that of the other nine commercial varieties are “A” (Figure 2a, Table 1 and Supplementary Table S2). It can be used to distinguish HuangJinGuo, ChuanHuangJin, HongYang and other 9 commercial varieties. Genetic distance analysis discovered that for *ITS2*, there is no difference among HuangJinGuo, ChuanHuangJin and HongYang. The genetic distance between them and other 9 commercial varieties was 0.01. There is no difference among the nine commercial varieties (Supplementary Table S5a). *ITS2* showed more variable sites than the plastid regions *matK* and *rbcL*. This result confirmed that *ITS2* showed greater discriminatory power than plastid regions [35]. Phylogenetic analysis also showed that HuangJinGuo, ChuanHuangJin and HongYang were clustered into one group with the *ITS2* sequence, and the other nine commercial varieties were grouped into another category (Figure 3a).

*rpl32_trnL*(*UAG*) had a greater degree of variation and could be used as the core barcode sequence of cherry plants [17]. *rpl32_trnL*(*UAG*) sequence analysis of 12 commercial kiwifruit results showed that compared to *rbcL*, *matK*, *psbA-trnH*, *rpoB*, *rpoC1*, *trnL* and *ITS2*, the *rpl32_trnL*(*UAG*) sequence difference is the maximum. It was found that there are six SNPs in the 1010bp of *rpl32_trnL*(*UAG*) sequence. Bases at 285 bp and 385 bp of CuiYu are “A”, that of the other 11 commercial varieties are “T”. Bases at 662 bp of HongYang are “C”, that of the other 11 commercial varieties are “T”. Bases at 768 bp of YaTe are “G”, that of the other 11 commercial varieties are “A”. Bases at 837 bp and 841 bp of HuaYou are “C” and “T”, respectively, that of the other 10 commercial varieties are “A”. Bases at 852 bp of XuXiang are “T”, that of the other 11 commercial varieties are “A”. (Figure 2c, Table 1 and Supplementary Table S4). It can be used to distinguish CuiYu, JinYan, YaTe, XuXiang, HuaYou, HongYang, HuangJinGuo and ChuanHuangJin from the other four commercial varieties, respectively. Genetic distance analysis discovered that, for *rpl32_trnL*(*UAG*), the genetic distance between HuaYou and JinYan, CuiXiang, FengXianLou, ChuanHuangJin, HaiWoDe, QinMei and HuangJinGuo is 0.002. The genetic distance between HongYang and CuiYu is 0.003. The genetic distance between HongYang and YaTe, XuXiang is 0.002. The genetic distance between HongYang and JinYan, CuiXiang, FengXianLou, ChuanHuangJin, HaiWoDe, QinMei and HuangJinGuo is 0.001. The genetic distance between CuiYu and YaTe, XuXiang is 0.003. The genetic distance between CuiYu and JinYan, CuiXiang, FengXianLou, ChuanHuangJin, HaiWoDe, QinMei and HuangJinGuo is 0.001. The genetic distance between JinYan and YaTe, XuXiang is 0.001. There is no difference between JinYan and CuiXiang, FengXianLou, ChuanHuangJin, HaiWoDe, QinMei and HuangJinGuo. The genetic distance between CuiXiang and YaTe, XuXiang is 0.001. There is no difference between CuiXiang and FengXianLou, ChuanHuangJin, HaiWoDe, QinMei and HuangJinGuo. The genetic distance between YaTe and XuXiang is 0.002. There is no difference between YaTe and FengXianLou, ChuanHuangJin, HaiWoDe, QinMei and HuangJinGuo. The genetic distance between FengXianLou and XuXiang is 0.001. There is no difference between FengXianLou and ChuanHuangJin, HaiWoDe, QinMei and HuangJinGuo. The genetic distance between ChuanHuangJin and XuXiang is 0.001. There is no difference between ChuanHuangJin and HaiWoDe, QinMei and HuangJinGuo. The genetic distance between HaiWoDe and XuXiang is 0.001. There is no difference between HaiWoDe and QinMei and HuangJinGuo. The genetic distance between QinMei and XuXiang is 0.001. There was no difference between QinMei and HuangJinGuo. The genetic distance between HuangJinGuo and XuXiang is 0.001 (Supplementary Table S5c). *rpl32_trnL*(*UAG*) showed more variable sites than the other plastid regions *matK* and *rbcL*. This result confirmed that *rpl32_trnL*(*UAG*) showed better discriminatory power than other plastid regions. Phylogenetic analysis further indicated that HuaYou, YaTe, XuXiang, HongYang, CuiYu can be distinguished from the other seven commercial varieties, respectively (Figure 3c).

Multi-site combination methods based on plastid (chloroplast) sequences are considered as effective strategies in the identification of plant species [31,33,34]. Therefore, we comprehensively analyzed the sequences of *ITS2*, *matK* combined with *rpl32_trnL*(*UAG*), and then found that, for *ITS2*+*matK+rpl32_trnL*(*UAG*), there is no difference between QinMei and YaTe, JinYan, XuXiang. There is no difference between HaiWoDe and YaTe, JinYan, XuXiang, QinMei. There is no difference between CuiXiang and YaTe, JinYan, XuXiang, QinMei, HaiWoDe. The genetic distance between YaTe and JinYan is 0.001. The genetic distance between XuXiang and YaTe, JinYan is 0.001. The genetic distance between HuaYou and YaTe, JinYan, XuXiang, QinMei, HaiWoDe, CuiXiang are 0.001. There is no difference between FengXianLou and YaTe, JinYan, XuXiang, QinMei, HaiWoDe and CuiXiang. The genetic distance between FengXianLou and HuaYou is 0.001. The genetic distance between CuiYu and YaTe, JinYan, XuXiang, HuaYou is 0.002. The genetic distance between CuiYu and QinMei, HaiWoDe, CuiXiang, FengXianLou is 0.001. The genetic distance between HuangJinGuo and YaTe, JinYan, XuXiang, HuaYou, CuiYu is 0.003. The genetic distance between HuangJinGuo and QinMei, HaiWoDe and CuiXiang, FengXianLou is 0.002. The genetic distance between HongYang and YaTe, JinYan, XuXiang, QinMei, HaiWoDe, CuiXiang, HuaYou, FengXianLou is 0.003. The genetic distance between HongYang and CuiYu is 0.004. There is no difference between HongYang and CuiYu. The genetic distance between ChuanHuangJin and YaTe, JinYan, XuXiang, HuaYou, CuiYu is 0.003. The genetic distance between ChuanHuangJin and QinMei, HaiWoDe and CuiXiang, FengXianLou is 0.002. There is no difference between ChuanHuangJin and HuangJinGuo, HongYang (Supplementary Table S5d). These results suggest that CuiYu, JinYan, YaTe, XuXiang, HuaYou, HongYang, HuangJinGuo and ChuanHuangJin can also be differentiated by the genetic distance of *ITS2*+*matK+rpl32_trnL*(*UAG*) combination. We found that the genetic distance between samples of HaiWoDe, FengXianLou, QinMei and CuiXiang in this study is 0.000, which means that most of them have a unique *ITS2*, *matK* and *rpl32_trnL*(*UAG*) sequences. This feature is thus useful for identifying these twelve commercial varieties and related species, which is similar to previous study [36].

The analysis of SNP loci is an important tool for analyzing small differences between populations [37,38,39]. In this study, by analyzing the sequence variable sites of 8 DNA barcodes including *ITS2*, *psbA-trnH*, *rbcL*, *matK*, *rpoB*, *rpoC1* and *rpl32_trnL*(*UAG*) in 12 commercial kiwifruit, stable SNP loci of DNA barcode *ITS2* are found between HuangJinGuo, ChuanHuangJin, HongYang and the other nine commercial varieties. Likewise, DNA barcode *matK* has stable SNP loci between JinYan, CuiYu and the other 10 commercial varieties. *rpl32_trnL*(*UAG*) has stable SNP loci between CuiYu, JinYan, YaTe, XuXiang, HuaYou, HongYang, HuangJinGuo and ChuanHuangJin and the other four commercial varieties. The presence of these SNP loci makes the identification of CuiYu, JinYan, YaTe, XuXiang, HuaYou, HongYang, HuangJinGuo and ChuanHuangJin kiwifruits possible. These results indicate that intergenic region *ITS2* could distinguish HuangJinGuo and HongYang from the nine other commercial varieties, and *matK* could be utilized to distinguish JinYan and CuiYu from 10 the other commercial varieties. *rpl32_trnL*(*UAG*) could be utilized to distinguish HuaYou, YaTe, XuXiang, HongYang, CuiYu from the other seven commercial varieties. SNPs of *ITS2*, *matK* and *rpl32_trnL*(*UAG*) combining with the current plant DNA barcode systems could be used to accurately identify kiwifruit, which comprises samples of closely-related or subspecies from different cultivation areas. These results are good agreements with previous reports [38,39].

In conclusion, morphologically, JinYan, CuiYu, HuangJinGuo and HongYang can be discriminated. At the molecular level, SNPs based on DNA barcoding intergenic region *ITS2* and cp gene *matK* and *rpl32_trnL*(*UAG*) combined together can distinguish CuiYu, JinYan, YaTe, XuXiang, HuaYou, HongYang, HuangJinGuo and ChuanHuangJin from the other four commercial varieties, respectively, that is consistent with the results of morphology and better than the results of morphology. These results will provide an experimental basis for the classification and identification of commercially available kiwifruits and will offer a theoretical support for scientific trials and forensic botany of such disputes.

## 4. Materials and Methods

### 4.1. Samples Collection and DNA Extraction

Seventy two samples from twelve kiwifruit commercial varieties were collected in this study. Their names are HuangJinGuo, CuiXiang, QinMei, XuXiang, HuaYou, FengXianLou, YaTe, HaiWoDe, CuiYu, ChuanHuangJin, HongYang and JinYan. Six replicates were randomly selected for each commercial variety in the local market. According to our survey, the price of HongYang is 4 to 5 times than that of QinMei, HuaYou, YaTe and HaiWoDe in the market. Similarly, ChuanHuangJin, HuangJinGuo and JinYan, whose price are 3 to 4 times than that of QinMei, HuaYou, YaTe and HaiWoDe. HuangJinGuo, CuiXiang, QinMei, XuXiang, HuaYou, FengXianLou, YaTe and HaiWoDe were collected from Shaanxi province of China. CuiYu, ChuanHuangJin, HongYang and JinYan were collected from Si Chuan province of China (Detailed information in Supplementary Table S6). Among the 12 commercial varieties, HongYang is diploid, HaiWoDe is hexaploid, HuangJinGuo, CuiXiang, XuXiang, HuaYou, CuiYu, ChuanHuangJin and JinYan are tetraploid. QinMei, FengXianLou and YaTe are unknown, which is needed further research. DNA of each kiwifruit was extracted by Plant Genomic DNA Extraction Kit (Omega, Norcross, GA, USA) according to the manufacturer’s instructions.

### 4.2. PCR Amplification, Cloning, and Sequencing

PCR primers of DNA barcodes (*rbcL*, *matK*, p*sbA-trnH*, *ITS2*, *rpoB*, *rpoC1*, *ycf1b*, *trnL*, *rpl32_trnL(UAG)* and *At103*) were designed based on DNA barcodes [9,10,11,16], CBOL Plant Working Group (2009), China Bole Group (2011) as well as reported chloroplast and genomic sequences of kiwifruit [40,41,42,43]. Primer sequences are listed in Table 1. They were synthesized in BGI (Beijing Genomics Institute, Beijing, China). Polymerase chain reaction made use of the same reaction system. Amplification procedures of ten different primer pairs are shown in Table 1. The PCR products were separated by 1.2~1.5% agarose gel electrophoresis. The remaining PCR products were recovered and purified. Then, the purified products were ligated into pMD18-T vector (Takara, DaLian, China) and transformed into DH-5α *E. coli.* Positive clones were screened and sequenced in Beijing Genomics Institute.

### 4.3. Sequence Alignments and Analysis

Multiple sequence alignment was performed by using the results of *psbA-trnH*, *rbcL*, *rpoB*, *rpoC1* and *trnL* amplification by http://multalin.toulouse.inra.fr/multalin/. (Supplementary Figure S1). Multi-sequence alignment of *ITS2*, *matK*, *rpl32_trnL*(*UAG*) and *ITS2*+*matK*+*rpl32_trnL*(*UAG*) were carried out by ClustalX 1.8 [44] software (Conway Institute UCD, Dublin, Ireland ), respectively. Then, the phylogenetic trees of these twelve kiwifruit commercial varieties were reconstructed. Neighbor-joining (NJ) method of MEGA 7.0 is applied to construct the phylogenetic tree. One thousand bootstrap resamplings were performed to assess the reliability. The steps are as follows: Phylogeny/construct/Test Neighbor-joining tree/Nucleotide Sequences/Statistical method: Neighbor-joining, No. of bootstrap repetitions: 1000, Model: Kimura 2-parameter model, Missing Data Treatment: Pairwise deletion/compute. The K2P distances between different genetic resources were calculated by MEGA7.0. Variation of different commercial varieties was analyzed according to the genetic distance of each barcode fragment.

## Figures and Tables

**Table 1:** SNP site information for two DNA barcodes.

Variety	SNP Site Information (bp)													
	*ITS2*					*matK*		*rpl32*						
	115	132	206	215	310	534	777	285	385	662	768	837	841	852
HuangJinGuo	C	C	G	G	C	A	A	T	T	T	A	A	A	A
ChuanHuangJin	C	C	G	G	C	A	A	T	T	T	A	A	A	A
HongYang	C	C	G	G	C	A	A	T	T	C	A	A	A	A
CuiiXiang	T	T	A	A	T	A	A	T	T	T	A	A	A	A
QinMei	T	T	A	A	T	A	A	T	T	T	A	A	A	A
JinYan	T	T	A	A	T	G	A	T	T	T	A	A	A	A
CuiYu	T	T	A	A	T	A	C	A	A	T	A	A	A	A
HaiWoDe	T	T	A	A	T	A	A	T	T	T	A	A	A	A
YaTe	T	T	A	A	T	A	A	T	T	T	G	A	A	A
FengXianLou	T	T	A	A	T	A	A	T	T	T	A	A	A	A
HuaYou	T	T	A	A	T	A	A	T	T	T	A	C	T	A
XuXiang	T	T	A	A	T	A	A	T	T	T	A	A	A	T

**Table 2:** Primers and amplification procedures used for PCR in this study.

Gene Amplification Region	Primer Name	Sequence (5′ →3′)	Length of Amplified Fragment	Amplification Procedure
*rbc*L	*rbcL*-F *rbcL*-R	ATGTCACCACAAACAGA TCGCATGTACCTGCAGTA	743 bp	94 °C 5 min; [94 °C 30S, 54.5 °C 30S, 72 °C 45S]* 35 cycles; 72 °C 10 min; 4 °C 10 min
*mat*K	*matK*-F *matK*-R	CGTACAGTACTTTTGTGTTTAC ACCCAGTCCATCTGGAAATC	889 bp	94 °C 5 min; [94 °C 30S, 56 °C 30S, 72 °C 55S]* 35 cycles; 72 °C 10 min; 4 °C 10 min
*psb*A*-trn*H	*psbA-trnH*-F *psbA-trnH*-R	TATGCATGAACGTAATGCT GCATGGTGGATTCACAAT	502 bp	94 °C 5 min; [94 °C 30S, 55 °C 30S, 72 °C 30S]* 35 cycles; 72 °C 10 min; 4 °C 10 min
*ITS2*	*ITS2*-F *ITS2*-R	ATGCGATACTTGGTGTG GACGCTTCTCCAGACTA	491 bp	94 °C 5 min; [94 °C 30S, 55 °C 30S, 72 °C 30S]* 35 cycles; 72 °C 10 min; 4 °C 10 min
*rpoB*	*rpoB*-F *rpoB*-R	AAGTGCATTGTTGGAACTGG CCCAGCATCACAATTCC	512 bp	94 °C 5 min; [94 °C 30S, 55 °C 30S, 72 °C 35S]* 35 cycles; 72 °C 10 min; 4 °C 10 min
*rpoC1*	*rpoC1*-F *rpoC1*-R	CAAAGAGGGAAGAT TAAGCATATCTTGAGT	529 bp	94 °C 5 min; [94 °C 30S, 54.5 °C 30S, 72 °C 40S]* 35 cycles; 72 °C 10 min; 4 °C 10 min
*rpl32_trnL(UAG)*	*rpl32* *trnL(UAG)*	CAGTTCCAAAAAAACGTACTTC CTGCTTCCTAAGAGCAGCGT	1010 bp	94 °C 5 min; [94 °C 30S, 57.8 °C 30S, 72 °C 45S]* 35 cycles; 72 °C 10 min; 4 °C 10 min
*trnL*	*trnL-F* *trnL-R*	CGAAATCGGTAGACGCTACG CCATTGAGTCTCTGCACCTATC	193 bp	94 °C 5 min; [94 °C 30S, 56.5 °C 30S, 72 °C 30S]* 35 cycles; 72 °C 10 min; 4 °C 10 min
*ycf1b*	*ycf1b-F* *ycf1b-R*	TCTCGACGAAAATCAGATTGTTGTGAAT ATACATGTCAAAGTGATGGAAAA	No result	94 °C 5 min; [94 °C 30S, 56.5 °C 30S, 72 °C 45S]* 35 cycles; 72 °C 10 min; 4 °C 10 min 56.5 °C
*At103*	*At103-F* *At103-R*	CTTCAAGCCMAAGTTCATCTTCTA ATCATTGAGGTACATNGTMACATA	No result	94 °C 5 min; [94 °C 30S, 54 °C 30S, 72 °C 45S]* 35 cycles; 72 °C 10 min; 4 °C 10 min 56.5 °C

## Supplementary Materials

The following are available online at https://www.mdpi.com/1420-3049/24/5/888/s1, Figure S1: *psbA-trnH*, *rbcL*, *rpoB*, *rpoC1* and *trnL* sequences alignment of 12 commercial kiwifruits. Figure S2: Phylogenetic analysis of 12 kiwifruit commercial varieties. Table S1: The information of the experimental materials, Table S2: Phenotypic characteristics of twelve commercial varieties of kiwifruit, Table S3: *ITS2* sequence of 72 kiwifruit samples, Table S4: *matK* sequence of 72 kiwifruit samples. Table S5: *rpl32_trnL* (*UAG*) sequence of 72 kiwifruit samples. Table S6: Genetic distance of *ITS2*, *matK*, *rpl32_trnL*(*UAG*) and *ITS2*+*matK+rpl32_trnL*(*UAG*) sequences of 12 kiwifruit commercial varieties.
