# Supplementary material for: Morphology and Molecular Identification of Twelve Commercial Varieties of Kiwifruit
Source: Molecules. 2019 Mar 3;24(5):888. doi: 10.3390/molecules24050888 (PMC6429161; doi:10.3390/molecules24050888)
Supplement: Supplementary file 1 [file molecules-24-00888-s001.zip › Supplementary Figure 2_Phylogenetic analysis of 12 kiwifruit commercial varieties.pdf]

Supplementary Figure 2. Phylogenetic analysis of 12 kiwifruit commercial varieties. (a) Analysis of 72 *ITS2* sequence fragments, (b) Analysis of 72 *matK* sequence fragments, (c) Analysis of 72 *rpl32_trnL(UAG)* sequence fragments.

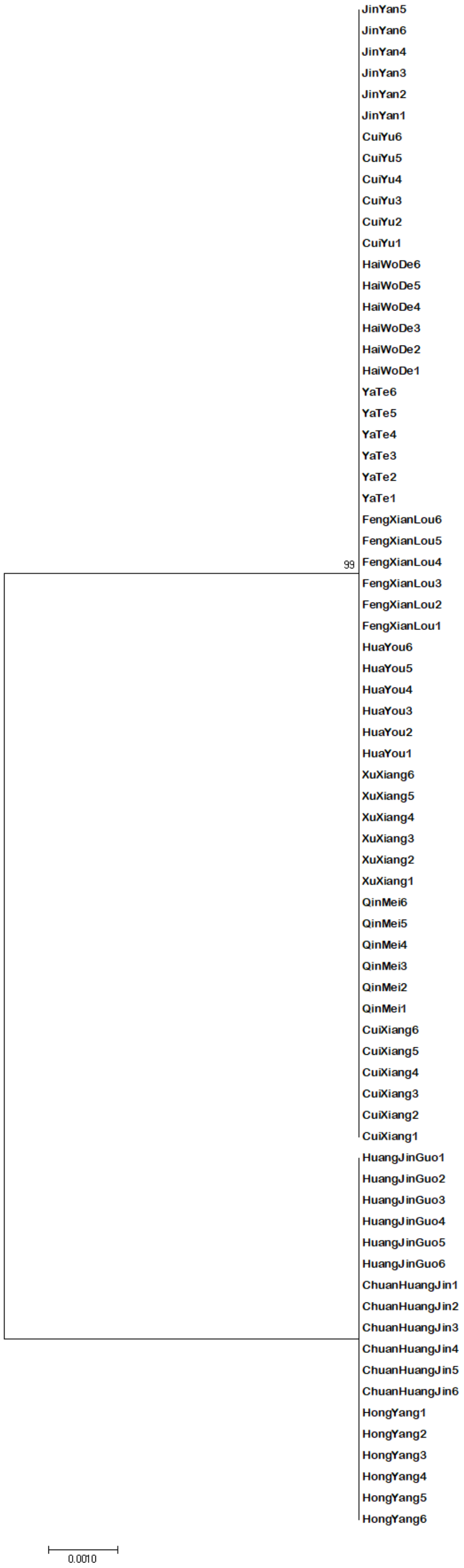

(a) Analysis of 72 *ITS2* sequence fragments

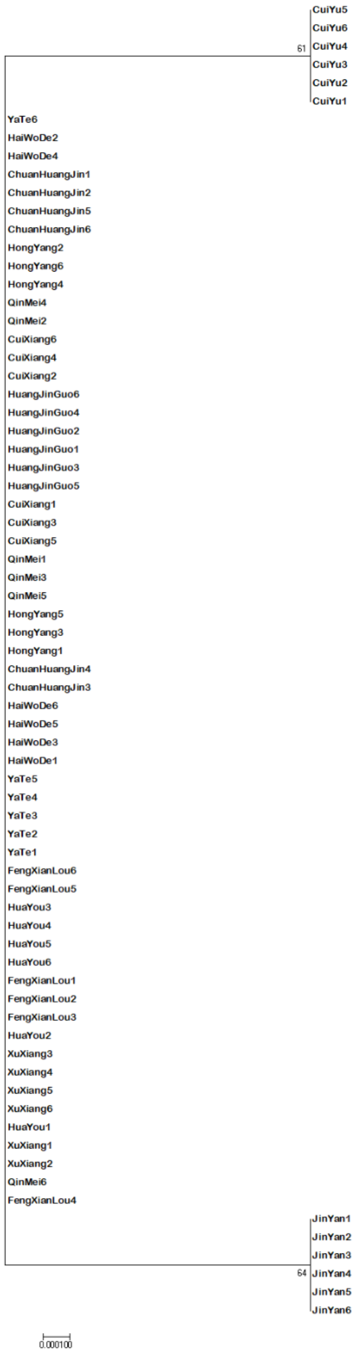

(b) Analysis of 72 *matK* sequence fragments

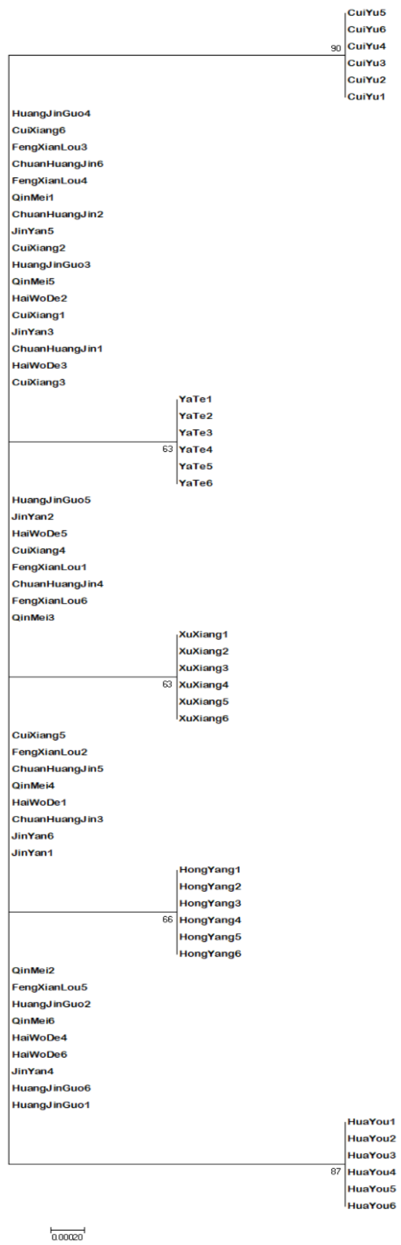

(c) Analysis of 72 *rpl32-trnL(UAG)* sequence fragments
