# Supplementary material for: Morphology and Molecular Identification of Twelve Commercial Varieties of Kiwifruit
Source: Molecules. 2019 Mar 3;24(5):888. doi: 10.3390/molecules24050888 (PMC6429161; doi:10.3390/molecules24050888)
Supplement: Supplementary file 1 [file molecules-24-00888-s001.zip › Supplementary Table 5_ genetic distance.pdf]

Supplementary Table 5a. Pairwise divergences of *ITS2* fragment between twelve kiwi commercial varieties

	CuiYu	JinYan	HaiWo De	YaTe	Feng Xian Lou	Hua You	Xu Xiang	Qin Mei	Cuii Xiang	Huang Jin Guo	Chuan Huang Jin	Hong Yang
CuiYu												
JinYan	0.000											
HaiWoDe	0.000	0.000										
YaTe	0.000	0.000	0.000									
FengXianLou	0.000	0.000	0.000	0.000								
HuaYou	0.000	0.000	0.000	0.000	0.000							
XuXiang	0.000	0.000	0.000	0.000	0.000	0.000						
QinMei	0.000	0.000	0.000	0.000	0.000	0.000	0.000					
CuiXiang	0.000	0.000	0.000	0.000	0.000	0.000	0.000	0.000				
HuangJinGuo	0.010	0.010	0.010	0.010	0.010	0.010	0.010	0.010	0.010			
ChuanHuangJin	0.010	0.010	0.010	0.010	0.010	0.010	0.010	0.010	0.010	0.000		
HongYang	0.010	0.010	0.010	0.010	0.010	0.010	0.010	0.010	0.010	0.000	0.000	

Supplementary Table 5b. Pairwise divergences of *matK* fragment between twelve kiwi commercial varieties

	Huang Jin Guo	Cui Xiang	Qin Mei	Xu Xiang	Hua You	Feng Xian Lou	Chuan Huang Jin	Hong Yang	HaiWo De	YaTe	JinYan	CuiYu
HuangJinGuo												
CuiXiang	0.000											
QinMei	0.000	0.000										
XuXiang	0.000	0.000	0.000									
HuaYou	0.000	0.000	0.000	0.000								
FengXianLou	0.000	0.000	0.000	0.000	0.000							
ChuanHuang Jin	0.000	0.000	0.000	0.000	0.000	0.000						
HongYang	0.000	0.000	0.000	0.000	0.000	0.000	0.000					
HaiWoDe	0.000	0.000	0.000	0.000	0.000	0.000	0.000	0.000				
YaTe	0.000	0.000	0.000	0.000	0.000	0.000	0.000	0.000	0.000			
JinYan	0.001	0.001	0.001	0.001	0.001	0.001	0.001	0.001	0.001	0.001		
CuiYu	0.001	0.001	0.001	0.001	0.001	0.001	0.001	0.001	0.001	0.001	0.002	

Supplementary Table 5c. Pairwise divergences of *rpl32_trnL*(*UAG*) fragment among twelve kiwi commercial varieties

	Hua You	Hong Yang	CuiYu	JinYan	Cui Xiang	YaTe	Feng Xian Lou	Chuan Huang Jin	HaiWo De	QinMei	Huang JinGuo	Xu Xiang
HuaYou												
HongYang	0.003											
CuiYu	0.004	0.003										
JinYan	0.002	0.001	0.002									
CuiXiang	0.002	0.001	0.002	0.000								
YaTe	0.003	0.002	0.003	0.001	0.001							
FengXianLou	0.002	0.001	0.002	0.000	0.000	0.001						
ChuanHuangJin	0.002	0.001	0.002	0.000	0.000	0.001	0.000					
HaiWoDe	0.002	0.001	0.002	0.000	0.000	0.001	0.000	0.000				
QinMei	0.002	0.001	0.002	0.000	0.000	0.001	0.000	0.000	0.000			
HuangJinGuo	0.002	0.001	0.002	0.000	0.000	0.001	0.000	0.000	0.000	0.000		
XuXiang	0.003	0.002	0.003	0.001	0.001	0.002	0.001	0.001	0.001	0.001	0.001	

Supplementary Table 5d. Pairwise divergences of *ITS2*+*matK*+*rpl32_trnL*(*UAG*) fragment among twelve kiwi commercial varieties

	YaTe	JinYan	Xu Xiang	QinM ei	HaiWo De	Cui Xiang	HuaY ou	Feng Xian Lou	CuiYu	Huang Jin Guo	Hong Yang	Chuan Huang Jin
YaTe												
JinYan	0.001											
XuXiang	0.001	0.001										
QinMei	0.000	0.000	0.000									
HaiWoDe	0.000	0.000	0.000	0.000								
CuiXiang	0.000	0.000	0.000	0.000	0.000							
HuaYou	0.001	0.001	0.001	0.001	0.001	0.001						
FengXianLou	0.000	0.000	0.000	0.000	0.000	0.000	0.001					
CuiYu	0.002	0.002	0.002	0.001	0.001	0.001	0.002	0.001				
HuangJinGuo	0.003	0.003	0.003	0.002	0.002	0.002	0.003	0.002	0.003			
HongYang	0.003	0.003	0.003	0.003	0.003	0.003	0.003	0.003	0.004	0.000		
ChuanHuangJin	0.003	0.003	0.003	0.002	0.002	0.002	0.003	0.002	0.003	0.000	0.000	
