# Supplementary material for: Morphology and Molecular Identification of Twelve Commercial Varieties of Kiwifruit
Source: Molecules. 2019 Mar 3;24(5):888. doi: 10.3390/molecules24050888 (PMC6429161; doi:10.3390/molecules24050888)
Supplement: Supplementary file 1 [file molecules-24-00888-s001.zip › Supplementary Table 6_The information of the experimental materials.pdf]

Supplementary Table 6. The information of the experimental materials.

serial number		Variety	source	Sequence length (bp) *ITS2*	Sequence length (bp) *matK*	*Sequence length (bp) rpl32_trnL(UAG)*	haplotype
1	1-1	HuangJinGuo	ZhouZhi, Shaanxi	491	889	1010	A1
2	1-2	HuangJinGuo	ZhouZhi, Shaanxi	491	889	1010	A1
3	1-3	HuangJinGuo	ZhouZhi, Shaanxi	491	889	1010	A1
4	1-4	HuangJinGuo	ZhouZhi, Shaanxi	491	889	1010	A1
5	1-5	HuangJinGuo	ZhouZhi, Shaanxi	491	889	1010	A1
6	1-6	HuangJinGuo	ZhouZhi, Shaanxi	491	889	1010	A1
7	2-1	CuiXiang	ZhouZhi, Shaanxi	491	889	1010	B1
8	2-2	CuiXiang	ZhouZhi, Shaanxi	491	889	1010	B1
9	2-3	CuiXiang	ZhouZhi, Shaanxi	491	889	1010	B1
10	2-4	CuiXiang	ZhouZhi, Shaanxi	491	889	1010	B1
11	2-5	CuiXiang	ZhouZhi, Shaanxi	491	889	1010	B1
12	2-6	CuiXiang	ZhouZhi, Shaanxi	491	889	1010	B1
13	3-1	QinMei	ZhouZhi, Shaanxi	491	889	1010	B1
14	3-2	QinMei	ZhouZhi, Shaanxi	491	889	1010	B1
15	3-3	QinMei	ZhouZhi, Shaanxi	491	889	1010	B1
16	3-4	QinMei	ZhouZhi, Shaanxi	491	889	1010	B1
17	3-5	QinMei	ZhouZhi, Shaanxi	491	889	1010	B1
18	3-6	QinMei	ZhouZhi, Shaanxi	491	889	1010	B1
19	4-1	XuXiang	ZhouZhi, Shaanxi	491	889	1010	C1
20	4-2	XuXiang	ZhouZhi, Shaanxi	491	889	1010	C1
21	4-3	XuXiang	ZhouZhi, Shaanxi	491	889	1010	C1
22	4-4	XuXiang	ZhouZhi, Shaanxi	491	889	1010	C1
23	4-5	XuXiang	ZhouZhi, Shaanxi	491	889	1010	C1
24	4-6	XuXiang	ZhouZhi, Shaanxi	491	889	1010	C1
25	5-1	HuaYou	ZhouZhi, Shaanxi	491	889	1010	D1
26	5-2	HuaYou	ZhouZhi, Shaanxi	491	889	1010	D1
27	5-3	HuaYou	ZhouZhi, Shaanxi	491	889	1010	D1
28	5-4	HuaYou	ZhouZhi, Shaanxi	491	889	1010	D1
29	5-5	HuaYou	ZhouZhi, Shaanxi	491	889	1010	D1
30	5-6	HuaYou	ZhouZhi, Shaanxi	491	889	1010	D1
31	6-1	FengXianLou	MeiXian, Shaanxi	491	889	1010	B1
32	6-2	FengXianLou	MeiXian, Shaanxi	491	889	1010	B1
33	6-3	FengXianLou	MeiXian, Shaanxi	491	889	1010	B1
34	6-4	FengXianLou	MeiXian, Shaanxi	491	889	1010	B1
35	6-5	FengXianLou	MeiXian, Shaanxi	491	889	1010	B1
36	6-6	FengXianLou	MeiXian, Shaanxi	491	889	1010	B1

serial number		Variety	source	Sequence length (bp) *ITS2*	Sequence length (bp) *matK*	Sequence length (bp) *rpl32*	haplotype
37	7-1	YaTe	MeiXian, Shaanxi	491	889	1010	E1
38	7-2	YaTe	MeiXian, Shaanxi	491	889	1010	E1
39	7-3	YaTe	MeiXian, Shaanxi	491	889	1010	E1
40	7-4	YaTe	MeiXian, Shaanxi	491	889	1010	E1
41	7-5	YaTe	MeiXian, Shaanxi	491	889	1010	E1
42	7-6	YaTe	MeiXian, Shaanxi	491	889	1010	E1
43	8-1	HaiWoDe	ZhouZhi, Shaanxi	491	889	1010	B1
44	8-2	HaiWoDe	ZhouZhi, Shaanxi	491	889	1010	B1
45	8-3	HaiWoDe	ZhouZhi, Shaanxi	491	889	1010	B1
46	8-4	HaiWoDe	ZhouZhi, Shaanxi	491	889	1010	B1
47	8-5	HaiWoDe	ZhouZhi, Shaanxi	491	889	1010	B1
48	8-6	HaiWoDe	ZhouZhi, Shaanxi	491	889	1010	B1
49	9-1	CuiYu	PuYang, SiChuan	491	889	1010	F1
50	9-2	CuiYu	PuYang, SiChuan	491	889	1010	F1
51	9-3	CuiYu	PuYang, SiChuan	491	889	1010	F1
52	9-4	CuiYu	PuYang, SiChuan	491	889	1010	F1
53	9-5	CuiYu	PuYang, SiChuan	491	889	1010	F1
54	9-6	CuiYu	PuYang, SiChuan	491	889	1010	F1
55	10-1	ChuanHuangJin	PuYang, SiChuan	491	889	1010	A1
56	10-2	ChuanHuangJin	PuYang, SiChuan	491	889	1010	A1
57	10-3	ChuanHuangJin	PuYang, SiChuan	491	889	1010	A1
58	10-4	ChuanHuangJin	PuYang, SiChuan	491	889	1010	A1
59	10-5	ChuanHuangJin	PuYang, SiChuan	491	889	1010	A1
60	10-6	ChuanHuangJin	PuYang, SiChuan	491	889	1010	A1
61	11-1	HongYang	PuYang, SiChuan	491	889	1010	G1
62	11-2	HongYang	PuYang, SiChuan	491	889	1010	G1
63	11-3	HongYang	PuYang, SiChuan	491	889	1010	G1
64	11-4	HongYang	PuYang, SiChuan	491	889	1010	G1
65	11-5	HongYang	PuYang, SiChuan	491	889	1010	G1
66	11-6	HongYang	PuYang, SiChuan	491	889	1010	G1
67	12-1	JinYan	PuYang, SiChuan	491	889	1010	H1
68	12-2	JinYan	PuYang, SiChuan	491	889	1010	H1
69	12-3	JinYan	PuYang, SiChuan	491	889	1010	H1
70	12-4	JinYan	PuYang, SiChuan	491	889	1010	H1
71	12-5	JinYan	PuYang, SiChuan	491	889	1010	H1
72	12-6	JinYan	PuYang, SiChuan	491	889	1010	H1
