# Supplementary figures and images for: Morphology and Molecular Identification of Twelve Commercial Varieties of Kiwifruit
Source: Molecules. 2019 Mar 3;24(5):888. doi: 10.3390/molecules24050888 (PMC6429161; doi:10.3390/molecules24050888)

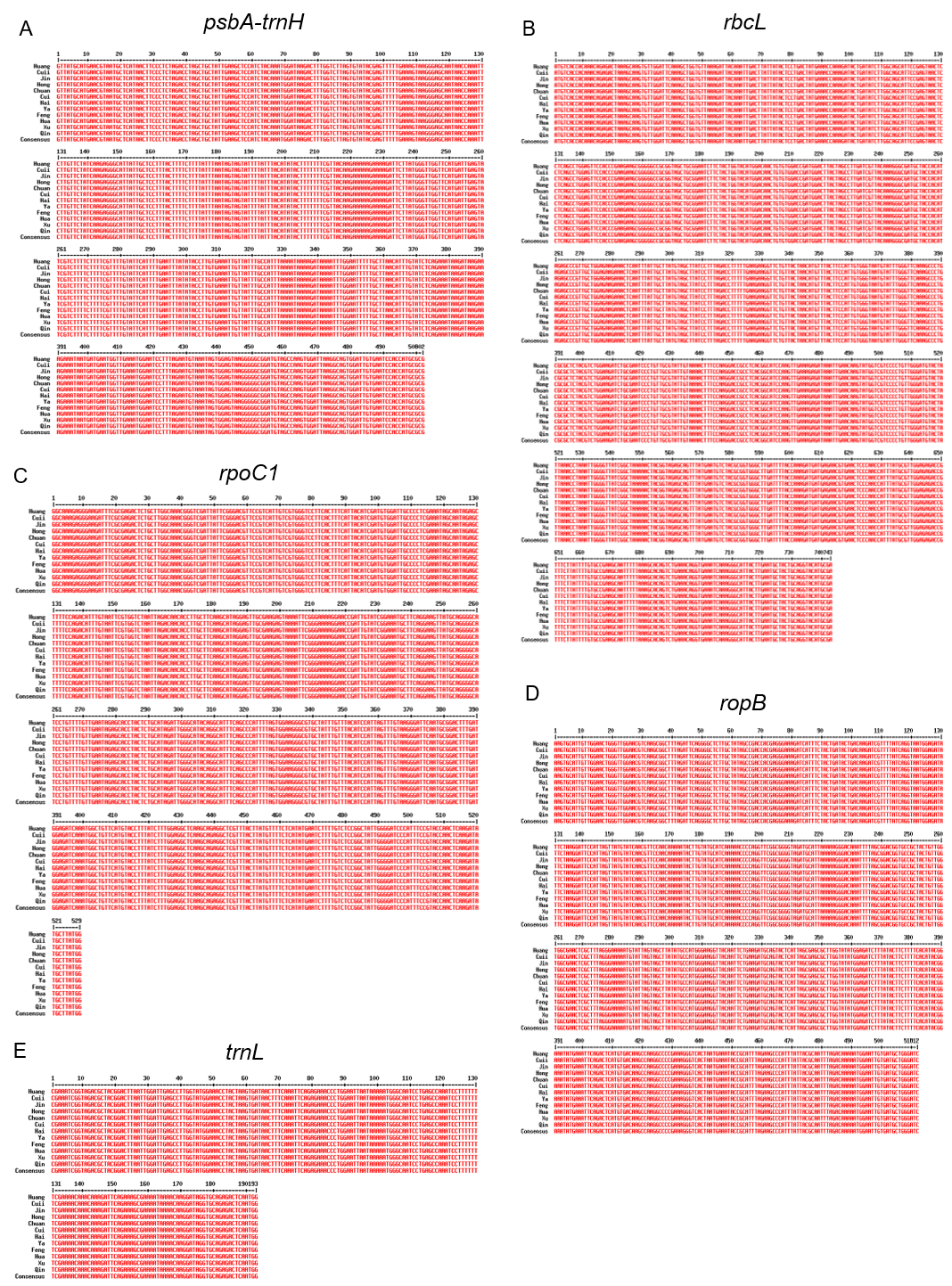

Supplement: Supplementary file 1 [file molecules-24-00888-s001.zip › Supplementary Figure 1.tif]
